# Citrus/Cydonia Comp. Can Restore the Immunological Balance in Seasonal Allergic Rhinitis-Related Immunological Parameters In Vitro

**DOI:** 10.1155/2008/496467

**Published:** 2008-11-30

**Authors:** E. W. Baars, H. F. J. Savelkoul

**Affiliations:** ^1^Department of Healthcare and Nutrition, Louis Bolk Instituut, 3972 LA Driebergen, The Netherlands; ^2^Department of Care, University of Applied Sciences Leiden, 2333 CK Leiden, The Netherlands; ^3^Department of Cell Biology and Immunology, Wageningen University, 6700 AH Wageningen, The Netherlands

## Abstract

In two in vitro studies, we examined the immunological (pathways of the) effects of Citrus/Cydonia comp. from, respectively, a healthy and an allergic donor; peripheral blood mononuclear cells (PBMCs) were isolated out of peripheral blood and analyzed in vitro after polyclonal stimulation of T-cells. The differentiation capacity and the influence with regard to Th1 (IFN-*γ*) and Th2 (IL-5) cells were examined. Citrus/Cydonia comp. has a selective effect on the differentiation of T-cells by producing relatively more IL-10 than IL-12. By that, it also seems to have an effect on the induction of regulatory (IL-10 producing) T-cell subsets. It is in vitro capable of neutralizing (to some extent) the changes, characteristic to allergic rhinitis, with regard to the maturation, differentiation, and activity of the immune system. Thus, Citrus/Cydonia comp. can potentially restore the disturbed immune state of rhinitis patients, which essentially could be sufficient to make allergic symptoms disappear permanently.

## 1. INTRODUCTION

Allergic rhinitis is a condition characterized by sneezing, watery nasal discharge, and nasal obstruction and itching. It is an increasingly prevalent condition, particularly in the Western world where it affects around 20% of the adult population. Allergic rhinitis is divided into seasonal allergic rhinitis (hay fever) which is triggered by pollens and moulds, and perennial allergic rhinitis in which house dust mites and pet dander are the predominant triggers. The spectrum of severity is wide and includes a significant number of sufferers with severe symptoms that are resistant to treatment with usual pharmacotherapy (antihistamines and topical nasal corticosteroids) [1]. The mean prevalence of allergic rhinitis in several Western countries is 12% [2].

Seasonal allergic rhinitis or hay fever is a type I immediate hypersensitivity reaction mediated by specific IgE antibody to a seasonal allergen, leading to mucosal inflammation characterized by sneezing, itching, rhinorrhoea, and nasal blockage. Pollens (6–40 *μ*m in diameter) from wind-pollinated grasses, trees, weeds, and spores from fungi are the most common aeroallergens. Grass pollen is the most common cause of seasonal allergic rhinitis. The highest levels of pollen in the atmosphereare found in May to July and pollen concentrations of 50 grains/mm^3^ are associated with symptoms in all susceptible people. The treatment of choice of seasonal allergic rhinitis is the symptomatic treatment with local or oral antihistamines and/or local corticosteroids. Immunotherapy, including sublingual immunomodulation therapy, is indicated in a limited subpopulation of patients [3].

Citrus/Cydonia comp. is an anthroposophic medicine, which contains extracts of lemon (Citrus lemon) and quince (Cydonia oblongata) [4]. For over eighty years now, the medicine “Citrus/Cydonia comp.” is being prescribed as a subcutaneous injection or as a nasal spray for patients who suffer from seasonal allergic rhinitis. A survey on clinical experiences, carried out among a group of 39 active Dutch general practitioners [4], indicates that the subcutaneous treatment with Citrus/Cydonia comp. ampoules is profoundly effective. Firstly, a permanent effect from the treatment with Citrus/Cydonia comp. tends to be experienced, which indicates that the patients in question are claiming to lastingly suffer less from hay fever or even that they are free from complaints. Secondly, the effect is occurring within a period of two weeks, up to three months, after the actual treatment. Thirdly, the effect is optimal after a treatment of several years. Moreover, the survey pointed out that preventive administration before the start of the pollen season with Citrus/Cydonia comp. may be even more efficacious to the patients in question. Recently, positive effects by Citrus/Cydonia comp. were obtained among a group of 13 patients with the following characteristics: (a) allergic to grass pollen, (b) suffering from hay fever, on average, for nine years, and (c) the necessity for the use of antihistamines with regard to the nature of the complaints [5]. In addition, a prospective, observational study on the effect of Citrus/Cydonia comp. nasal spray on hay fever symptoms reported positive results without side effects in 140 patients [6]. We now performed in vitro studies to study the possible immunological (pathways of the) effects of Citrus/Cydonia comp.

## 2. MATERIALS AND METHODS

### 2.1. Blood donors

From a healthy and an allergic individual, 8 mL of blood was collected in sodium heparinate-coated vacutainers (BD Biosciences, San Diego, Calif, USA). The allergic individual was sensitized for birch pollen (RAST 6+) and grass pollen (RAST 4+), and he was also food allergic with a positive skin prick test on apple and cherry. The blood was subsequently diluted 1:1 with IMDM containing GlutaMAX (IMDM; Gibco-BRL, Paisley, Scotland) before the density gradient centrifugation on Ficoll-Paque PLUS (Amersham Biosciences, Uppsala, Sweden). The PBMC layer was washed twice with IMDM and the cell viability and cell concentration were determined by Trypan blue exclusion. An informed consent was obtained before the sample collection and the performed experiments were approved by the local ethical committee.

### 2.2. Culture conditions

PBMCs were cultured in Yssel’s medium at 37°C in a humidified atmosphere with 5% CO_2_ at a density of 1⋅10^6^ viable cells/mL. Yssel’s medium consisted of IMDM supplemented with 1% Penicillin-Streptomycin (Gibco BRL), extra additions according to Yssel et al. [1984], and 1% human AB serum (Gibco BRL). Cells were plated out in 48 well plates at a concentration of 1⋅10^6^ cells/mL and cultured at 37°C. After five hours of adaptation to the culture conditions, the various stimuli or a matching volume of medium were added. Cultures were stimulated with 150 ng/mL anti-CD3 plus 100 ng/mL anti-CD28monoclonal antibodies (BD Pharmingen, San Diego, Calif, USA) or cultured in medium only [7].

### 2.3. Citrus/Cydonia comp. stimulation

Both conditions (negative and positive controls) took place in the presence of Citrus/Cydonia comp. 100 *μ*L/1 mL culture in two dilutions (undiluted and 1:3 dilution in culture medium). The extract (Gencydo) was obtained from (Weleda, Zoetermeer, The Netherlands).

### 2.4. Cell viability

Half a million PBMCs were washed and subsequently incubated with 2 *μ*L Annexin V-APC (BD Biosciences) in 200 *μ*L Annexin V buffer according to the manufacturer’s protocol. After an incubation period of 15 minutes on ice, the cells were spun down (400 g for 10 minutes) and resuspended in 200 *μ*L Annexin V buffer and 2 *μ*L PI (1 mg/mL; Sigma, St. Louis, Mo, USA). The cells were then analyzed on a flow cytometer (FACS array, BD Biosciences).

### 2.5. Immunological phenotype

The immunological phenotype of PBMC subsets was determined by staining the surface antigens with the following two monoclonal antibody (*α*) mixtures: (1) *α*-hCD3 (PE-Cy7), *α*-hCD4 (PE), *α*-hCD8 (APC), and *α*-hCD25 (APC-Cy7); (2) *α*-hCD3 (PE-Cy7), *α*-hCD14 (APC), *α*-hCD16 (PE), *α*-hCD19 (APC-Cy7), and *α*-hCD56 (PE). All antibodies were purchased at BD Biosciences.

Per well, 5⋅10^5^ cells were spun down in a 96 wells U-bottom plate. The cells were incubated with staining buffer (1% FCS and 0.1 M NaN_3_ in PBS) containing the surface markers or the matching isotype controls for 30 minutes on ice in the dark. The cells were washed once with PBS and resuspended in PBS for flow cytometry. The four-color flow cytometric acquisition was performed on an FACS array, using the BD FACS-array software. An electronic gate was set to exclude debris and at least 10 000 events/samples were acquired. The percentages of positive cells were corrected for the isotype control.

### 2.6. Proliferation capacity

The proliferation capacity of the PBMC was studied by intracellular expression of the nuclear Ki-67 antigen (Ki-67; BD Pharmingen). The Ki-67 antigen is absent in the nuclei of resting cells, but present in all other phases of the cell division cycle as well as in the mitosis phase [8]. In each well, 5⋅10^5^ PBMCs were incubated with 100 *μ*L Cytofix/Cytoperm (BD Pharmingen) for 15–20 minutes on ice to fix and permeabilize the cells. Cells were washed twice with perm/wash buffer (BD Pharmingen) and incubated with anti-Ki-67 PE antibody, or the matched isotype control, diluted in perm/wash buffer for 30 minutes on ice in the dark. Hereafter, the cells were washed with perm/wash buffer, resuspended in PBS, and measured on the flow cytometer. Values are expressed as cells positive for the Ki-67 mAb corrected for the isotype control.

### 2.7. Cytokines

PBMC culture supernatants were analyzed for their IL-1*β*, IL-12, IFN-*γ*, TNF-*α*, IL-4, IL-5, IL-10, and IL-13 contents. The cytokine production was measured with Cytometric Bead Assay Flex Sets (BD Pharmingen). All buffers used in this protocol were obtained from the BD CBA Soluble Protein Master Buffer Kit (BD Pharmingen). Supernatants were collected, stored at −20°C, and tested within 2 weeks. The procedure was performed according to the manufacturer’s protocol. The samples were measured on the FACS array, using the FCAP software. The sensitivity limits for quantitative determinations, according to the manufacturer, were 1.1 pg/mL for IL-1*β*, 0.3 pg/mL for IL-4 and IFN-*γ*, 0.5 pg/mL for IL-5, 2.3 pg/mL for IL-10, 2.2 pg/mL for IL-12, 0.6 pg/mL for IL-13, and 0.7 pg/mL for TNF-*α*.

## 3. RESULTS

### 3.1. Effect of Citrus/Cydonia comp. on healthy donor PBMC

PBMCS were isolated and analyzed for their subset composition. The percentages of the subsets PBMC were 63% CD3+ T-cells (with 50.4% CD4+ Th-cells and 12.6% CD8+ Tc-cells), 8% CD19+ B cells, 5% CD14+ monocytes, and 12% CD16/CD56+ NK cells. After one day (Table 1) and four days (Table 2) of culture, the results showed that Citrus/Cydonia comp. not only induced T-cell proliferation directly, but also activated monocytes resulting in a selective cytokine production (TNF-*α*, IL-1*β*, IL-10, and IL-12). However, Citrus/Cydonia comp. induced more IL-10 than IL-12 production most likely derived from monocytes, thereby stimulating the outgrowth of immunoregulatory (IL-10) monocytes more than the immunoreactive (IL-12) subsets of monocytes. These monocyte-related effects of Citrus/Cydonia comp. were detectable within one day and were found in cultures with a normal therapeutic dose. Subsequently, T-cell activation and proliferation were suprastimulated by Citrus/Cydonia comp. over the polyclonal stimulation alone. Citrus/Cydonia comp. had no effect on cell survival and did appear to be toxic for PBMC cell subpopulations.

### 3.2. Effect of Citrus/Cydonia comp. on an allergic donor PBMC

After four days (Table 3), the results demonstrated that Citrus/Cydonia comp. was able to restore the reduced IL-10 production in PBMC cultures of the allergic individuals. The stronger immunoregulatory balance (IL-10) and a curbed augmented Th2 response (a decrease of IL-4 and IL-5 production) was accompanied by an increased production of IL-12 and a reduced production of IFN-*γ*. Even after 4 days, monocyte stimulation by Citrus/Cydonia comp. was detected by the production of TNF-*α* and IL-10.

## 4. DISCUSSION

Here, we show that Citrus/Cydonia comp. has a selective effect on the differentiation of T-cells with regard to the production of cytokines; the production of IL-10 is relatively larger than that of IL-12. By that, Citrus/Cydonia comp. also seems to have an effect on the induction of regulatory (IL-10 producing) T-cell subsets. Hence, as a consequence, Citrus/Cydonia comp. might be producing an allergy reducing effect, at which it does not concern Th1 induction and the reduction of the allergen-specific Th2 response, bearing the risk of induction of a chronic inflammation and very likely even an increased risk for autoimmunity.

Recent developments mainly concern the field of allergen-specific immunotherapeutic protocols. This immunotherapy is widely believed to occur through restoration of the disturbed Th1-Th2 balance [9–12], either linked to the induction of allergen-specific (blocking) IgG4 antibodies, or to the induction of regulatory T-cell subsets. The exact role which the regulatory T-cell subsets play with regard to these mechanisms is not yet indistinct. With regard to the development of these immunotherapeutic protocols, special allergen preparations, obtained from purified natural or recombinant produced allergens, are necessary and need to be developed.

Citrus/Cydonia comp. is likely to induce more regulatory T-cells, whether CD4+CD25+Fosp3+ natural or antigen-induced IL-10 and/or TGF-b producing Tr-cells, that are, therefore, very immunosuppressive, and which are capable of reducing allergen specifically activated Th2 cells. Our results imply that Citrus/Cydonia comp. does not induce a complete state of immunosuppression, resulting in a diminished resistance against infections and a reduced protection against tumors. This is consistent with the long-term clinical experiences and the results of the empirical studies on the use of Citrus/Cydonia comp.

Based on these in vitro investigations, we hypothesized that Citrus/Cydonia comp. is capable of neutralizing (to some extent) the changes, characteristic to allergic rhinitis, with regard to the construction, the maturation, the differentiation, and the activity of the immune system. By that, it is possible to explain the therapeutic positive effects on allergic rhinitis patients treated with Citrus/Cydonia comp. found in previous studies and clinical practice.

The conclusions based on this study are of great importance, since the standard treatment of allergic rhinitis is based on the long-term use of antihistamines, potentially in combination with a local application of corticosteroids, in case of persisting and/or serious symptoms. Those treatments tend to reduce the symptoms, but they do not possess any immunotherapeutic potency themselves. This implies that it is compulsory for individual patients to keep on using such medicines for many years. Based on our pilot data, indicating that in vitro Citrus/Cydonia comp. is capable of modulating the Th1-Th2 balance, we actually expect Citrus/Cydonia comp. to have an immunotherapeutic potency. This adds to the clinical therapeutic effect from Citrus/Cydonia comp., both as an injection and as a topical application. This implies that a long-term treatment with Citrus/Cydonia comp. injections during several years, before the start of the pollen season, can potentially restore the disturbed immune state of rhinitis patients, which essentially could be sufficient to make the allergic complaints disappear.

## Figures and Tables

**Table 1:** Mean scores after one day (healthy donor).

	Medium	Medium + Citrus/Cydonia comp. 1	Medium + Citrus/Cydonia comp. 1:3
Proliferation (%)	3 (1)*	13 (3)	1 (1)
Cell death (%)	88 (12)	82 (11)	87 (5)

Cytokines (pg/mL)

TNF-*α*	13 (4)	3315 (129)	13 (1)
IL-1*β*	25 (6)	3535 (147)	15 (2)
IL-10	22 (9)	918 (52)	12 (1)
IL-12	12 (3)	46 (12)	12 (2)
IFN-*γ*	15 (2)	55 (8)	25 (3)
IL-4	10 (2)	12 (1)	10 (1)
IL-5	12 (2)	28 (3)	10 (1)

Percentages of proliferating cells and cells in apoptosis of PBMC cultures of a healthy donor stimulated for one day with medium or Citrus/Cydonia undiluted and 1:3 diluted. In the supernatants of these cultures, cytokines were measured by flow cytometric analysis in the Bead Assay Flex Sets system. Cytokine levels in pg/mL. *All results are described with standard deviations (SDs).

**Table 2:** Mean scores after four days (healthy donor).

	Medium	Anti-CD3/28	Medium + Citrus/Cydonia comp. 1	Medium + Citrus/Cydonia comp. 1:3	Stimulation + Citrus/Cydonia comp. 1	Stimulation + Citrus/Cydonia comp. 1:3
Proliferation (%)	3 (1)*	42 (8)	3 (1)	1 (1)	48 (11)	40 (2)
Cell death (%)	88 (12)	61 (11)	87 (8)	92 (11)	65 (12)	60 (6)

Cytokines (pg/mL)

TNF-*α*	13 (2)	8483 (987)	15 (2)	13 (2)	9647 (733)	8117 (566)
IL-1*β*	15 (3)	4134	35 (11)	15 (1)	4858 (247)	4037 (138)
IL-10	12 (2)	9553	18 (8)	12 (2)	12276 (566)	5662 (931)
IL-12	12 (1)	84	26 (9)	12 (2)	238 (87)	185 (77)
IFN-*γ*	15 (2)	34355	15 (1)	15 (2)	48800 (12778)	37750 (12501)
IL-4	12 (2)	134	12 (1)	12 (2)	55 (12)	106 (28)
IL-5	12 (2)	375	12 (2)	12 (1)	118 (45)	266 (56)

Percentages of proliferating cells and cells in apoptosis of PBMC cultures of a healthy donor stimulated for four days with medium alone, polyclonal stimulation with anti-CD3 plus anti-CD28 antibodies, or polyclonal stimulation in the presence of Citrus/Cydonia preparation undiluted and 1:3 diluted. In the supernatants of these cultures, cytokines were measured by flow cytometric analysis in the Bead Assay Flex Sets system. Cytokine levels in pg/mL. *All results are described with standard deviations (SDs).

**Table 3:** Mean scores after four days (allergic donor).

	Medium	Stimulation with anti-CD3/28	Stimulation + Citrus/Cydonia comp.
Proliferation (%)	1 (1)*	48 (12)	57 (12)
Cell death (%)	78 (8)	59 (16)	55 (8)

Cytokines

TNF-*α*	10 (1)	5782 (154)	6893 (738)
IL-1*β*	10 (1)	2275 (339)	3772 (665)
IL-10	10 (1)	2331 (452)	7634 (1299)
IL-12	10 (1)	134 (26)	335 (89)
IFN-*γ*	10 (1)	9778 (452)	11668 (1638)
IL-4	10 (1)	456 (87)	138 (18)
IL-5	10 (1)	667 (154)	227 (85)

Percentages of proliferating cells and cells in apoptosis of PBMC cultures of an allergic donor stimulated for four days with medium alone, polyclonal stimulation with anti-CD3 plus anti-CD28 antibodies, or in the presence of Citrus/Cydonia undiluted preparation. In the supernatants of these cultures, cytokines were measured by flow cytometric analysis in the Bead Assay Flex Sets system. Cytokine levels in pg/mL. *All results are described with standard deviations (SDs).
